# Complete Genome Sequence of Lacticaseibacillus paracasei Strain VHProbi O44, Isolated from Feces from a Healthy Baby

**DOI:** 10.1128/mra.01071-22

**Published:** 2022-12-01

**Authors:** Qian Wang, Hongchang Cui, Chaoqun Guo, Zhi Duan

**Affiliations:** a Qingdao Vland Biotech Group Co., Ltd., Qingdao, China; University of Maryland School of Medicine

## Abstract

Lacticaseibacillus paracasei strain VHProbi O44 is a Chinese commercial lactic acid bacterium with several probiotic functions. The whole genome contains a chromosome and three plasmids.

## ANNOUNCEMENT

Infant fecal samples were collected in accordance with the Declaration of Helsinki, serially diluted with 0.85% (wt/vol) NaCl, and homogenized using a Stomacher. The dilution was spread onto De Man-Rogosa-Sharpe (MRS) agar (Luqiao, China) plates and incubated at 37°C for 48 h to enrich bacteria. A selected bacterial strain was named VHProbi O44. The bacterium was identified by Gram staining, biochemical and molecular biological analyses, such as with API 50 CHL medium, 16S rRNA gene Sanger sequencing, and mass spectrometry (1). The results showed that this strain belongs to the species Lacticaseibacillus paracasei. The Illumina HiSeq 2500 and Pacific Biosciences (PacBio) RS II sequencing platforms were combined for sequencing of this strain. Strain VHProbi O44 was cultured using the same method as for isolation to extract genomic DNA. Total DNA was extracted using a genomic DNA purification kit (Promega, USA) (2). DNA samples were quality controlled using a TBS-380 fluorometer (Turner BioSystems Inc.), and high-quality DNA was used for further research (3). Illumina template DNA was sheared into 400-bp fragments for library creation using the Illumina TruSeq library preparation kit. The prepared libraries were then used for paired-end Illumina sequencing (2 × 150 bp). For PacBio sequencing, DNA was centrifuged in g-TUBES (Covaris, Woburn, MA) for shearing into fragments. Then, ~10-kb fragments were purified, end repaired, and ligated with SMRTbell sequencing adapters following the manufacturer’s recommendations (PacBio, Menlo Park, CA) (4, 5). Next, an insert library was prepared using the SMRTbell Express template preparation kit v2.0 and sequenced in one single-molecule real-time (SMRT) Cell using standard methods. Finally, 7,024,462 raw reads and 6,801,162 clean reads were generated using the Illumina sequencing platform, to reach a depth of 337.42-fold coverage, and 87,960 raw reads (*N*_50_, 12,674 bp) were generated using the PacBio sequencing platform. Adapters, non-A/G/C/T bases at the 5′ end, reads containing >10% unknown N bases, and low-quality reads were removed by using Sickle v1.33 (6). Clean data were assembled into a scaffold using SOAPdenovo v2.04 (7). The PacBio raw reads were then assembled into a scaffold using the Hierarchical Genome Assembly Process (HGAP) and Canu v2.2 (8). Error correction of the PacBio assembly results was performed with Pilon v1.22 (9) using the Illumina reads. If there was a 5,000-bp overlap of the two ends, then one end of the overlap was cut off to circularize the sequence. The final assembly generated a complete genome with a seamless chromosome and three plasmids. Glimmer v3.02 (10), tRNAscan-SE v2.0 (11), and Barrnap v0.9 (https://github.com/tseemann/barrnap) were used for prediction of coding sequences (CDSs), tRNAs, and rRNAs, respectively. Genome annotations were conducted using the NCBI Prokaryotic Genome Annotation Pipeline (PGAP) v6.2 (12). Default parameters were used for all software unless otherwise specified.

The genome of Lacticaseibacillus paracasei strain VHProbi O44 consists of one circular chromosome (3,105,961 bp) and three plasmids (79,930 bp, 68,258 bp, and 64,133 bp), with GC contents of 46.24%. There were 3,096 protein-coding genes, 15 rRNA genes (5S rRNA, 16S rRNA, and 23S rRNA), 59 tRNA genes, 3 noncoding RNA genes, and 92 pseudogenes in the genome.

### Data availability.

The final annotated genome sequence of Lacticaseibacillus paracasei strain VHProbi O44 has been deposited in the GenBank database with accession numbers CP104303, CP104304, CP104305, and CP104306. The 16S rRNA gene sequence has been deposited in the GenBank database with accession number OP692715. The SRA accession numbers are SRR21285515 and SRR21285516. The BioProject accession number is PRJNA874474, and the BioSample accession number is SAMN30550144.
